# Enhanced therapeutic effect of PEDF-loaded mesenchymal stem cell-derived small extracellular vesicles against oxygen-induced retinopathy through increased stability and penetrability of PEDF

**DOI:** 10.1186/s12951-023-02066-z

**Published:** 2023-09-08

**Authors:** Ruiyan Fan, Lin Su, Hui Zhang, Yilin Jiang, Zihao Yu, Xiaomin Zhang, Xiaorong Li

**Affiliations:** Tianjin Key Laboratory of Retinal Functions and Diseases, Tianjin Branch of National Clinical Research Center for Ocular Disease, Eye Institute, School of Optometry, Tianjin Medical University Eye Hospital, Tianjin, 300384 China

**Keywords:** Mesenchymal stem cells, Small extracellular vesicles, Pigment epithelium-derived factor, Oxygen-induced retinopathy mouse model

## Abstract

**Background:**

Several common retinal diseases that cause blindness are characterised by pathological neovascularisation accompanied by inflammation and neurodegeneration, including retinopathy of prematurity (ROP), diabetic retinopathy (DR), age-related macular degeneration (AMD), and retinal vein occlusion (RVO). The current treatment strategies for these diseases have limited benefits. Thus, safer and more effective alternative approaches are required. In this study, we loaded small extracellular vesicles (sEVs) derived from mesenchymal stem cell (MSC) with pigment epithelium-derived factor (PEDF), and tested the therapeutic effect of PEDF-loaded sEVs (PEDF-sEVs) using an oxygen induced retinopathy (OIR) mouse model, aiming to establish a new therapy strategy for the treatment of retinal pathological angiogenesis.

**Results:**

We formulated PEDF-loaded sEVs (PEDF-sEVs) containing high concentrations of PEDF and evaluated their effects through in vivo and in vitro experiments. In OIR mice, PEDF-sEVs showed significantly better effects on retinal avascular areas, inflammation, and neuronal degeneration compared with the anti-vascular endothelial growth factor (VEGF) drug, which may indicate a possible advantage of PEDF-sEVs over anti-VEGF drugs in the treatment of pathological neovascularisation. In vitro, PEDF-sEVs greatly inhibited endothelial cell (EC) proliferation, migration, and tube formation by suppressing the VEGF-induced phosphorylation of extracellular signal-regulated kinase (ERK) and AKT (also known as Protein Kinase B). All experiments and analyses were performed in triplicate. PEDF-sEVs were more effective than PEDF or sEVs alone, both in vitro and in vivo. Furthermore, to determine the distribution of PEDF-sEVs, we used DiD-labelled sEVs and FITC-labelled PEDF to track the sEVs and PEDF, respectively. We found that PEDF-sEVs effectively reduced the degradation of PEDF.

**Conclusions:**

Loading PEDF on sEVs effectively enhanced the anti-angiogenic, anti-inflammatory, and neuroprotective effects of PEDF by increasing the stability and penetrability. These results suggest a potential role for PEDF-sEVs in retinal pathological neovascularisation.

**Graphical Abstract:**

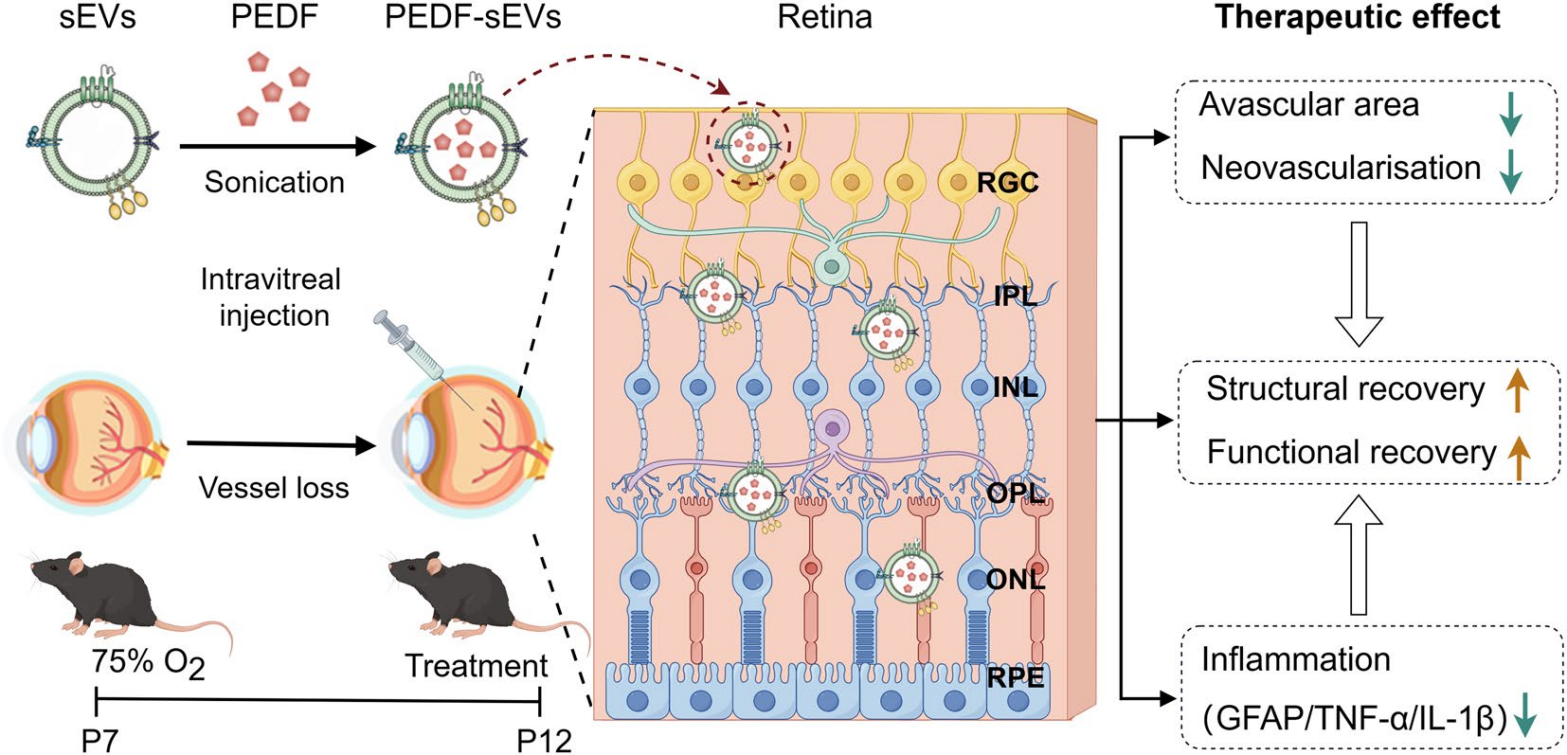

**Supplementary Information:**

The online version contains supplementary material available at 10.1186/s12951-023-02066-z.

## Background

Abnormal retinal or choroidal neovascularisation (NV) is a key pathological change in several common blinding retinal diseases, including retinopathy of prematurity (ROP) [1], diabetic retinopathy (DR) [2], age-related macular degeneration (AMD) [3], and retinal vein occlusion (RVO) [4]. Abnormal vessels are more vulnerable to bleeding, causing retinal and vitreous haemorrhage, retinal detachment, macular damage, and eventually blindness [5]. Additionally, these diseases are characterised by retinal inflammation and neuronal degeneration [6]. Current clinical treatments include intravitreal injections of anti-VEGF drugs, retinal photocoagulation, and vitrectomy [7–9]. Although an intravitreal injection of anti-VEGF drugs can inhibit the further formation of retinal and choroidal NV, their therapeutic effects have been disputed, as approximately 30% of patients respond poorly to anti-VEGF treatment [10–12]. Thus, alternative approaches that are more effective and safer are required.

PEDF, also known as early population doubling level cDNA-1, is a 50 kDa multifunctional member of the serine proteinase inhibitor (serpin) family [13–15]. It was originally isolated from the conditioned medium of cultured human foetal retinal pigment epithelial (RPE) cells [16]. Several subsequent studies have shown that PEDF is widely expressed in a variety of tissues and has multiple physiological effects, which include anti-angiogenic, anti-inflammatory, antioxidant, and neuroprotective effects [16–18]. However, PEDF has not been used in clinical practice due to its limited tissue penetrability and biological stability.

Exosomes are extracellular vesicles secreted by various types of cells. Typically, they have a diameter of 30–150 nm and contain proteins, DNA, RNA, amino acid metabolites, lipids, cytoplasm, and cell surface proteins [19]. Exosomes are important mediators of intercellular communication. We have previously shown that exosomes derived from MSCs exhibit properties similar to those of parent cells, such as anti-inflammatory, immuno-suppressive, and neuroprotective effects [20–22]. Unlike cells, exosomes cannot replicate, differentiate, or mutate. Exosomes derived from MSCs are considered ideal drug carriers because of their small particle size, low immunogenicity, non-toxicity, and biodegradability, and they can transfer various proteins and regulatory genes to tissues or cells [23, 24]. They can be stored at -80 °C for a long time [25]. Exosomes have been shown to enhance the efficacy of loaded drugs, such as interleukin 10 [26], rapamycin [27], human chorionic gonadotropin [28], catalase [29], triptolide [30], and paclitaxel [31, 32] by improving the stability of these drugs. Therefore, they are promising drug carriers for ophthalmic disease therapies.

To improve the therapeutic effects of PEDF in this study, we constructed MSC-derived exosomes highly loaded with PEDF and tested their therapeutic effects on oxygen-induced retinopathy (OIR) mice, an animal model of ROP, which is characterised by retinal neovascularisation, as well as inflammation and neuronal damage. According to the consensus of the International Society for Extracellular Vesicles in 2018, we have used the term sEVs instead of exosomes in this study, because the current separation protocols cannot completely remove all non-exosome vesicles [33]. Our results suggest that MSC-sEVs loaded with PEDF (PEDF-sEVs) are more effective than PEDF or sEVs alone in treating OIR mice by increasing the stability and penetrability of PEDF. In addition, PEDF-sEVs exhibited superior anti-inflammatory and neuroprotective effects compared with anti-VEGF drugs.

## Materials and methods

### Animals

C57BL/6J mice were purchased from specific pathogen-free Biotechnology Co., Ltd. (Beijing, China). All procedures involving mice were approved by the Animal Care and Use Committee of Tianjin Medical University Eye Hospital and conformed to the ARVO Statement for the Use of Animals in Ophthalmic and Vision Research (Permit Number: TJYY2019091225).

### Isolation and identification of MSCs

Human umbilical cord MSCs were obtained from Beijing Beilai Biological Co., Ltd. (China). Fresh human umbilical cords were obtained and enzyme-digested as previously described to isolate MSCs [27]. Briefly, the umbilical cords were washed twice, cut into approximately 1–3 mm^3^ sections and digested with 0.1% collagenase type II (17,101,015, Gibco, USA) at 37 °C for 1 h on a shaker. The cell suspension was then filtered through a mesh and centrifuged. The cell precipitate was re-suspended in complete Dulbecco’s modified Eagle’s medium/nutrient mixture F12 (C11330500BT, DMEM/F12; Gibco) complete medium. The medium contained 10% foetal bovine serum (16,000,044, FBS; Gibco), 100 U/mL penicillin, and 100 mg/mL streptomycin. The cells were seeded in T175 flasks and cultured at 37 °C in a 5% CO_2_ incubator. The adipogenic, osteogenic, and chondrogenic differentiation abilities of MSCs were evaluated using stem cell differentiation kits according to the manufacturer’s instructions (90,021, 90,031, and 90,041, respectively; OriCell, China).

### Isolation and identification of sEVs

Passage 3–5 MSCs were cultured with 25 mL culture medium containing 10% sEV-free FBS (prepared using ultracentrifugation at 110,000 × *g* at 4 °C overnight) for 48 h. Supernatants were collected from MSC culture and sEVs were isolated by ultracentrifugation. The supernatants were centrifuged at 2000 × *g* for 10 min and then at 10,000 × *g* for 30 min to remove cellular debris and dead cells. This was followed by two rounds of ultracentrifugation (Beckman Coulter, USA) at 110,000 × *g* for 70 min at 4 °C. The pellets were resuspended in 300 μL PBS and sterilised by filtration through a pre-rinsed 0.22 mm filter. Protein concentrations in the isolated sEVs were quantified using a Bicinchoninic Acid (BCA) protein assay kit (PC0020, Solarbio, China). The size distribution of sEVs derived from MSCs was determined using nanoparticle tracking analysis (NTA) (NanoSight NS300, Malvern, UK). The morphology of sEVs was visualised using high-resolution transmission electron microscopy (TEM; Hitachi HT7700, Japan). Twenty milligrams of sEVs were used for western blotting to detect the typical sEV markers, including CD9, CD63, and tumour susceptibility 101 (TSG101).

### Preparation of PEDF-sEVs

PEDF-sEVs were prepared by sonication. PEDF (1177-SF-025, R&D SYSTEMS, USA) was mixed with sEVs at a 1:5 concentration ratio. The mixture was incubated for 10 min and sonicated using an ultrasonic cell crusher (Fisher Scientific* Model 120, Hampton, NH, USA) on ice (20% power, 45 cycles of a 4 s pulse/2 s pause). The mixture was incubated at 37 °C for 1 h to allow the recovery of the sEV membrane. The unloaded PEDF was removed by centrifugation at 14,000 × *g* for 20 min in a 100 kDa diafiltration tube (UFC510096, Millipore, Germany). The final solution was collected by reversing the tube and centrifuging at 2000 × *g* for 2 min, and was used to test the loading capacity and encapsulation efficiency using a PEDF ELISA kit (RD191114200R, R&D SYSTEMS) following the manufacturer’s instructions. The drug-loading capacity (LC) was calculated according to the following formula: Loading Capacity (%) = (Drug entrapped)/(Drug entrapped + Total mass of sEVs) × 100. The encapsulation efficiency (EE) was calculated as follows: Encapsulation efficiency (%) = (Drug entrapped)/(Total amount of drug) × 100. All experiments were performed in triplicate.

The isolated sEVs were labelled with DiD (V22887, Invitrogen) following the manufacturer’s instructions. FITC-labelled PEDF was synthesised by CLOUD-CLONE CORP (RPB972Hu01, Wuhan, China). PEDF_FITC_-sEVs prepared using sonication were used to measure the loading efficiency on a FACSCalibur flow cytometer (BD Biosciences, USA), and the data were analysed using flow cytometry software (FlowJo, USA). All experiments were performed in triplicate.

### Endothelial cell proliferation assay

Human retinal endothelial cells (HRECs), purchased from the American Type Culture Collection (ATCC), were cultured in Endothelial Cell Medium (1001, ECM, ScienCell, USA) containing 5% FBS, 1% endothelial cell growth supplements, and 1% antibiotic solution. All cells were used from passage 2 to 6. For stimulation, the cells were starved in ECM supplemented with 2.5% FBS, 0.5% endothelial cell growth supplements, and 1% antibiotic solution.

A cell proliferation assay was performed using the Cell Counting Kit-8 (CCK-8; HY-K0301, MedChemExpress, USA). HRECs were seeded at 4 × 10^3^ cells/well in 96-well plates, allowed to adhere for 12 h, and then incubated with VEGF (10 ng/mL; 48,143, Cell Signalling Technology, UK), PEDF (400 ng/mL), sEVs (10 µg/mL), a mixture of PEDF (400 ng/mL) and sEVs (10 µg/mL) without sonication, or PEDF-sEVs (10 µg/mL) in starvation medium. After 24 h of incubation, cells were incubated with a mixture containing 90 µL ECM and 10 µL CCK-8 reagent for 2–4 h at 37 °C. Absorbance was measured at 450 nm using a multimode microplate reader (Infinite M200 Pro, Thermo Fisher Scientific). All experiments were performed in triplicate.

### Transwell assay

Transwell chambers (3422, Corning Incorporated, USA) were used for the migration assay. HRECs (8 × 10^4^) were seeded in the upper chamber in FBS-free medium in 24-well plates and incubated with VEGF, PEDF, sEVs, a mixture of PEDF and sEVs without sonication, or PEDF-sEVs. The bottom of the chamber contained the ECM medium supplemented with 10% FBS. After the cells had migrated for 24 h, they were fixed and stained with crystal violet. The migrated HRECs were counted under an inverted light microscope. The number of migrated HRECs was quantified by counting the cells in five random fields. All experiments were performed in triplicate.

### Scratch assay

HRECs (1.5 × 10^5^) were seeded in 12-well plates. After 12 h, a wound was made by scraping the cell monolayer with a 1 mL pipette tip, and cells were stimulated with VEGF, PEDF, sEVs, a mixture of PEDF and sEVs without sonication, or PEDF-sEVs for 24 h in the starvation medium. Images were acquired at 0 and 12 h after incubation at 37 °C. The area of wound closure between 0 and 12 h was analysed using ImageJ software. All experiments were performed in triplicate.

### Tube formation assay

Forty-eight-well plates were pre-coated with 150 µL Matrigel (Bedford, USA) for 30 min at 37 °C. Then, 3 × 10^4^ HRECs per well were seeded on Matrigel (354,234, Biocoat, USA) and treated with VEGF, PEDF, sEVs, a mixture of PEDF and sEVs without sonication, or PEDF-sEVs in the starvation medium. The images were acquired after 4 h. The nodes, master conjunctions, and lengths of the tubes were measured using ImageJ software. All experiments were performed in triplicate.

### Analysis of VEGF downstream signalling in HRECs

To detect VEGF downstream signalling, 8 × 10^4^ HRECs were seeded in 12-well plates for 12 h and then treated with PEDF, sEVs, or PEDF-sEVs for 24 h. The HRECs were then stimulated with or without VEGF (10 ng/mL) for 20 min. The cells were washed with PBS and lysed in cold RIPA lysis buffer supplemented with 1% PMSF (P0100, Solarbio) and 1% phosphatase inhibitor (P1260, Solarbio) for western blotting to detect the relative expression of ERK, p-ERK, AKT, and p-Akt. All experiments and analyses were performed in triplicate.

### Application of MSC-sEVs in oxygen-induced retinopathy mouse model

An oxygen-induced retinopathy mouse model (OIR) was established, as previously described [34]. Briefly, neonatal C57BL/6J mice and nursing mothers were exposed to hyperoxia (75% O_2_) on postnatal day 7 (P7) for 5 days and returned to room air on P12. The number of neonatal mice assigned to each nursing mother was the same. The mice were provided with a standard diet and water and randomly assigned to each of the treatment groups. 0.4% oxybuprocaine hydrochloride eye drops were used for topical anesthesia of OIR mouse eyes before intravitreal injection. The OIR mice were treated with 1 µL of PBS, PEDF (40 µg /mL), sEVs (1 mg/mL), a mixture of PEDF (40 µg /mL) and sEVs (1 mg/mL) without sonication, anti-VEGF drug (10 mg/mL, Ranibizumab, Genentech, USA) or PEDF-sEVs (the protein concentration of sEVs was 1 mg/mL) through intravitreal injection on P12. A 10 µL 34G Hamilton syringe (Hamilton, Reno, NV, USA) was used for intravitreal injections. The mice were euthanised on P17, and the retinas were dissected along the cornea–sclera divide. The iris, cornea, lens, and vitreous were removed and discarded. Retinas were peeled off, immediately flash-frozen in liquid nitrogen, and then stored at − 80 °C for western blot and PCR analyses.

### Quantification of avascular area and neovascularisation

OIR mice were sacrificed on P17. The eyeballs were enucleated and fixed in 4% paraformaldehyde (p1110, PFA, Solarbio) for 30 min. Retinas were dissected, washed with PBS, permeabilised with PBS containing 1% TritonX-100 for 30 min, and then blocked in PBS containing 2% BSA, 0.3% TritonX-100 at 4 °C for 2 h. Subsequently, flat-mounted retinas were stained with isolectinGS-IB4 (121,411, 1:500, Thermo Fisher Scientific) overnight at 4 °C in the dark for visualisation of retinal vasculature in OIR mice. Retinal vascular structures were observed using a confocal fluorescence microscope (LSM 800; Carl Zeiss, Germany). According to previously described methods [34], the areas of vaso-obliteration and retinal neovascular tufts were quantified using the Adobe Photoshop software (Adobe, USA).

### Immunofluorescence

On P17, OIR mice were sacrificed. Eyeballs from each group were enucleated, transferred to an optimal cutting temperature compound (4583, OCT, Solarbio) and frozen at − 80 °C. Sections were cut at a thickness of 8 μm and fixed in 4% PFA for 30 min. For immunofluorescence staining of glial fibrillary acidic protein (GFAP), the sections were incubated overnight in a blocking solution with the anti-GFAP antibody (ab7260, 1:500, Abcam, UK) in a humidified chamber at 4 °C. After washing, the sections were incubated with Alexa Fluor 594-conjugated goat anti-rabbit immunoglobulin G (ab150080, 1:2000, Abcam) at room temperature (RT) for 3 h in the dark. The nuclei of the retinal cells were stained with DAPI (C0065, Solarbio). Finally, the sections were observed under a confocal fluorescence microscope (LSM 800; Carl Zeiss), and the fluorescence intensity was analysed using ImageJ software.

### Optical coherence tomography imaging

Spectral domain optical coherence tomography (OCT; Heidelberg Engineering) imaging was performed on P25 (± 1 d) and P42 (± 1 d) to evaluate the retinal thickness. Mice were anaesthetised, and their pupils were dilated using Tropicamide Eye Drops (Alcon, Belgium). The retinas were scanned using an OCT camera placed in front of the cornea, with the optic disc positioned at the centre of the image. OCT image data were collected using a computer and retinal thickness was automatically quantified using the OCT software.

### Electroretinogram

Electroretinogram (ERG) was performed on P25 (± 1 d) and P42 (± 1 d) to evaluate retinal function of OIR mice using Ganzfeld Electroretinogram (Phoenix Micron IV, Phoenix Technologies, USA). The mice were subjected to dark adaptation overnight and were anaesthetised before the ERG. Corneas were anaesthetised with 0.4% oxybuprocaine hydrochloride, and Gatifloxacin Eye Gel was applied to the ocular surface to prevent tissue dryness. The reference electrode was inserted under the scalp, close to the midline between the ears. Another electrode was inserted into the mouse tail. Pupils were centred to the camera and a range of light intensity stimulation parameters of − 1.1, 0.1, 1.0, and 3.0 log (cd•s/ m^2^) were set for waveform detections. Each mouse was tested three times to obtain an average value. The a-wave amplitude was measured from the baseline to the trough of the a-wave, and the b-wave amplitude was measured from the trough of the a-wave to the peak of the b-wave, which was automatically quantified using the ERG software.

### Haematoxylin-eosin staining

Haematoxylin-eosin (H&E) staining was performed on P25 and P42 to evaluate retinal structures in OIR mice. Eyes from each group were fixed, dehydrated, and embedded in paraffin. Then, the paraffin-embedded eyes were sectioned at a thickness of 4 μm and stained with H&E (G1120, Solarbio). The stained sections were examined under a microscope.

### Toxicity assessment

OIR mice were sacrificed on P17. The eyeballs from each group were enucleated and transferred to an optimal cutting temperature compound. Sections were cut at a thickness of 8 μm and fixed in 4% PFA for 30 min. A FragEL™ DNA Fragmentation Detection Kit (11,684,817,910, Roche Molecular Biochemicals, Germany) was used to evaluate the toxicity of PEDF-sEVs on the retina. DAPI was used to stain cell nuclei. Finally, sections were observed under a confocal fluorescence microscope (LSM 800, Carl Zeiss). Terminal deoxynucleotidyl transferase-mediated dUTP nick end labelling (TUNEL)-positive cells in the retina were counted to quantify toxicity [35].

### PEDF-sEVs uptake by HRECs

DiD-labelled sEVs and FITC-labelled PEDF were used to test the cellular uptake of sEVs by HRECs. Synthesised FITC-labelled PEDF were obtained from CLOUD-CLONE CORP. HRECs (1.5 × 10^4^) were seeded in 24-well plates for 12 h, and then treated with 10 µg/mL PEDF_FITC_-sEVs or a mixture of PEDF (400 ng/mL) and sEVs (10 µg/mL) for 24 or 48 h. The concentration of PEDF was consistent with that of the PEDF-sEVs, as measured using ELISA. Subsequently, the cells were washed with PBS, fixed with 4% PFA at RT for 30 min, and nuclei were stained with DAPI. The cells were imaged using a confocal microscope (LSM800, Carl Zeiss). All experiments were performed in triplicate.

To compare the efficiency of PEDF delivery in HRECs, cells from each group were harvested. Flow cytometry data were collected using a FACSCalibur flow cytometer (BD Biosciences, USA) and analysed using flow cytometry software (FlowJo, USA). All experiments were performed in triplicate.

### Detection of PEDF concentration in supernatants collected from HRECs

HRECs (4 × 10^3^) were seeded in 96-well plates for 12 h. Subsequently, they were treated with PEDF (400 ng/mL), sEVs (10 µg/mL), or PEDF-sEVs (10 µg/mL) for 6, 24, 48, or 72 h. The supernatants were collected and lysed in cold RIPA lysis buffer with 1% PMSF (P0100, Solarbio) and 1% phosphatase inhibitor (P1260, Solarbio) to measure the concentration of PEDF using a PEDF ELISA kit (R&D SYSTEMS), following the manufacturer’s instructions. All experiments were performed in triplicate.

### Ocular distribution of PEDF

FITC-labelled PEDF was used to detect the distribution of PEDF in the retina. OIR mice were treated with PEDF, sEVs, or PEDF-sEVs via intravitreal injections on P12. Mice were sacrificed on P13 or P17. The eyes from each group were enucleated and transferred to an optimal cutting temperature compound and frozen at − 80 °C. Sections were cut at a thickness of 8 μm and fixed in 4% PFA for 30 min. Then the sections were stained with DAPI for 5 min and observed under a confocal fluorescence microscope (LSM 800, Carl Zeiss). The fluorescence intensity of FITC was analysed using ImageJ software.

### RNA extraction and quantitative real-time PCR analysis

Total RNA was extracted from HRECs and retinas using a universal RNA Purification Kit (B004, EZ Bioscience, USA) following the manufacturer’s protocol. The concentration and quality of RNA were examined using Nanodrop 2000 (Thermo Fisher Scientific). RNA samples were reverse-transcribed to complementary DNA (cDNA) using a Colour Reverse Transcription Kit (A0010CGQ, EZBioscience). Quantitative polymerase chain reaction (qPCR) was performed using SYBR Green Master Mix (A0012, EZBioscience). GAPDH was used as an internal reference for each reaction. The relative expression was calculated using the following equation: Relative gene expression = 2^[△Ct(control)–△Ct(target)]^. The sequences of the primers are listed in Additional file 1: Table S1.

### Western blot analysis

Cells or retinal tissues were lysed in cold RIPA lysis buffer supplemented with 1% PMSF (P0100, Solarbio) and 1% phosphatase inhibitor (P1260, Solarbio) for 10 min, and the total protein concentration was measured using a BCA protein assay kit (PC0020, Solarbio). Proteins (20 µg for each sample) were subjected to electrophoresis on a 10% or 12.5% SDS-polyacrylamide gel for 1 h. The electrophoresed proteins were transblotted to a polyvinylidene fluoride membrane (RF1136, Millipore). Next, 5% non-fat milk or 5% BSA in Tris-buffered saline with Tween (TBST, T1082, Solarbio) buffer was used to block the membranes for 2 h at RT. The membranes were then incubated overnight with the following primary antibodies at 4 °C: anti-CD9 (ab92726, 1:1000, Abcam), anti-CD63 (ab216130, 1:2000, Abcam), anti-TSG101 (ab125011, 1:1000, Abcam), anti-pAkt (4060, 1:2000, Cell Signaling Technology), anti-Akt (4691, 1:2000, Cell Signaling Technology), anti-ERK (4370, 1:2000, Cell Signaling Technology), anti-pERK (4695, 1:2000, Cell Signaling Technology), anti-VEGF (ab46154, 1:1000, Abcam), anti-PEDF (ab180711, 1:1000, Abcam), anti-GFAP (ab7260, 1:5000, Abcam), anti-ICAM-1 (10831-1-AP, 1:1000, Proteintech), or anti-GAPDH (60004-1, 1:1000, Proteintech), which was used as an internal reference. The membranes were washed and incubated with the appropriate secondary antibody for 2 h at RT. Bound primary antibodies were detected using horseradish peroxidase-conjugated goat anti-mouse IgG (7076, 1:2000, Cell Signaling Technology) and goat anti-rabbit IgG (7074, 1:2000, Cell Signaling Technology). The processed blots were developed using Immobilon ECL reagent (RPN2232, Cytiva) and imaged using a transilluminator (Tanon, China). The membranes were then stripped in a Stripping Buffer (CW0056M, Cwbiotech, Shanghai, China) and re-probed with another primary antibody. Pixel densities of the protein bands were calculated using the ImageJ software, and protein expression values were divided by those of GAPDH.

### Statistical analysis

Data are presented as mean ± standard deviation (SD). To calculate statistical significance, a Student’s *t*-test was used for two-group comparisons, and one-way analysis of variance was used for multigroup comparisons. The GraphPad Prism 9.4 software (GraphPad Software, USA) was used for statistical analysis and mapping charts. A *p*-value ≤ 0.05 was considered to indicate a statistically significant difference.

## Results

### Identification of MSC-derived sEVs and loading efficiency of PEDF-sEVs

**Fig. 1:**
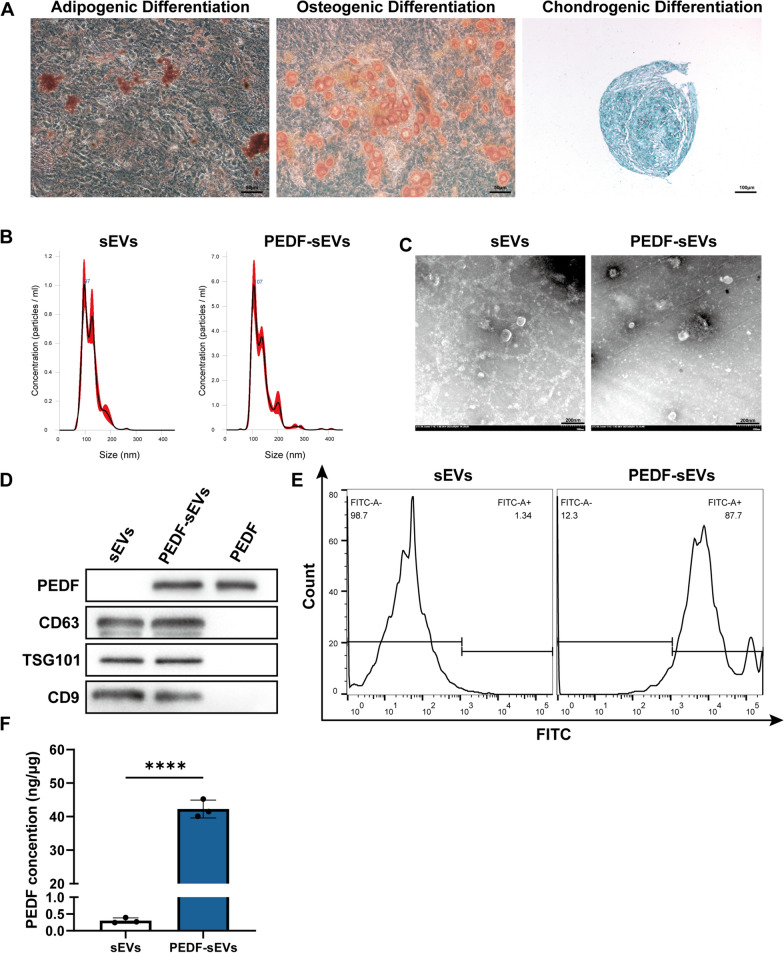
Characterisation of MSCs and sEVs. **A** Representative images of adipogenic (oil red O staining), osteogenic (alizarin red S staining), and chondrogenic (alcian blue staining) differentiation assay. **B** Analysis of sizes of sEVs from each group using Nanosight. **C** Representative transmission electron micrograph for each group; scale bar = 200 nm. **D** Representative western blots for loaded proteins and markers (PEDF, CD63, TSG101, and CD9) in MSC-sEVs. **E** Flow cytometry for measuring the loading efficiency of PEDF in PEDF-sEVs. FITC-labelled PEDF was used. **F** The concentrations of PEDF in sEVs and PEDF-sEVs were determined using ELISA (*n* = 3/group). The data are represented as mean ± SEM. *p < 0.05, **p < 0.01, ***p < 0.001, ****p < 0.0001

The surface antigens of MSCs were identified using flow cytometry, as previously described [26, 27]. MSCs were positive for CD73 and CD90 and negative for CD45 and CD34. Additionally, the differentiation of MSCs into adipocytes, osteoclasts, and chondrocytes in the differentiation medium was used to characterise their functional properties (Fig. 1A). The sizes of the sEVs were measured using Nanosight. The diameters of the sEVs exhibited a relatively narrow distribution and mainly ranged from 80 to 150 nm in the sEV and PEDF-sEV groups (Fig. 1B). TEM showed that the sEVs in both groups were typically cup-shaped, with a double-layer membrane structure (Fig. 1C). To test the feasibility of using PEDF-sEVs as a drug delivery system, we first isolated sEVs from the supernatants of MSCs by serial differential centrifugation and ultracentrifugation and examined their characteristics. Western blotting confirmed that these nanovesicles were positive for CD9, CD63, and TSG101, and that PEDF-sEVs contained PEDF (Fig. 1D). To evaluate whether PEDF can be efficiently loaded onto sEVs, we used FITC-labelled PEDF and performed flow cytometry. The results revealed that 87% of sEVs contained PEDF (Fig. 1E). Moreover, PEDF-sEVs contained a higher concentration of PEDF than sEVs, as indicated by the ELISA results (Fig. 1F). The drug loading capacity of PEDF in PEDF-sEVs was 4.08 ± 0.07%, and the encapsulation efficiency was 11.34 ± 0.93%. These data indicated that the PEDF-sEVs prepared by sonication exhibited a high drug-loading capacity.

### **PEDF-sEVs inhibit VEGF-induced HREC proliferation, migration, and tube formation** in vitro

**Fig. 2:**
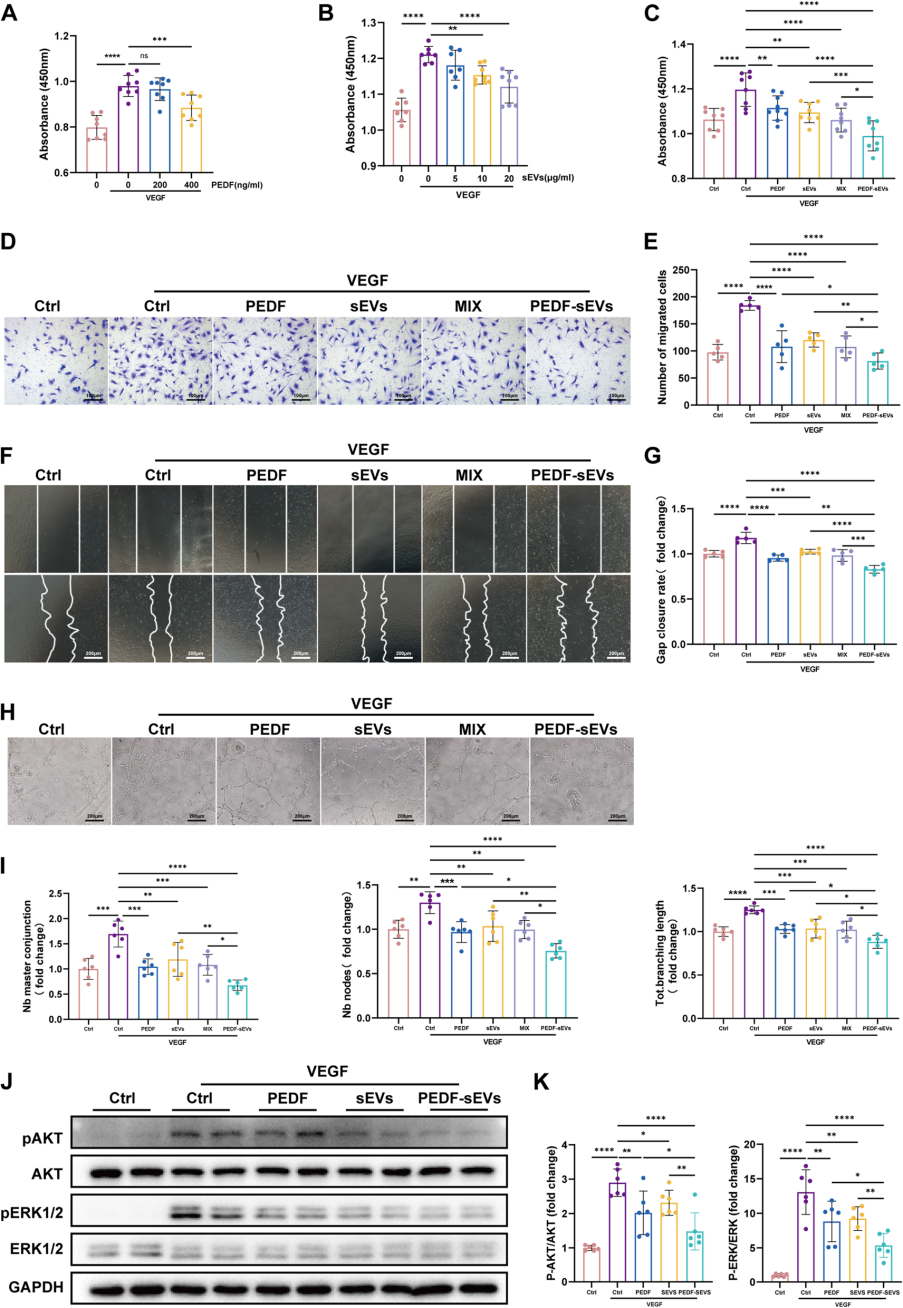
PEDF-sEVs suppress VEGF-induced angiogenic effects and VEGF-downstream signalling in HRECs. **A** Starved HRECs were treated with PEDF under VEGF stimulation for 24 h. Cell proliferation was measured using a CCK assay (OD = 450 nm, *n* = 8). **B** Starved HRECs were treated with sEVs under VEGF stimulation for 24 h. Cell proliferation was measured using a CCK assay (OD = 450 nm, *n* = 7). **C** Starved HRECs were treated with PEDF (400 ng/mL), sEVs (10 µg/mL), a mixture of PEDF (400 ng/mL) and sEVs (10 µg/mL) without sonication, or PEDF-sEVs (10 µg/mL) under VEGF (10 ng/mL) stimulation for 24 h. Cell proliferation was measured using a CCK assay (OD = 450 nm, *n* = 8). **D** Representative images and **E** Quantification of the Transwell assay of HRECs treated as in **C** after 24 h (*n* = 5); scale bar = 100 μm. **F ** Representative images and **G** Quantification of the scratch migration assay in HRECs treated as in **C** after 12 h (*n* = 5), scale bar = 200 μm. **H** Representative images and **I** Quantification of the tube formation assay in HRECs treated as in **C** after 2–4 h (*n* = 6), scale bar = 200 μm. **J** Starved HRECs were pre-treated with PEDF, sEVs, or PEDF-sEVs for 24 h and then stimulated with 10 ng/mL VEGF for 20 min. Representative western blots for pAKT, AKT, pERK1/2, and ERK1/2 showing VEGF-downstream signalling. **K** Quantitative analysis for qERK/ERK and qAKT/AKT in HRECs (*n* = 6). The data are represented as mean ± SEM. *p < 0.05, **p < 0.01, ***p < 0.001, ****p < 0.0001

To evaluate the effects of PEDF-sEVs on angiogenesis, we performed in vitro angiogenesis assays including endothelial cell (EC) proliferation, migration, and tube formation assays. The CCK-8 assay was used to test whether PEDF-sEVs directly affected the proliferation of HRECs. In our study, VEGF at a concentration of 10 ng/mL significantly increased EC proliferation. We investigated the inhibitory effects of different concentrations of PEDF (0, 200, and 400 ng/mL) on VEGF-stimulated HRECs. The proliferation of HRECs was significantly inhibited by 400 ng/mL PEDF (Fig. 2A). We also verified the effects of different concentrations (0, 5, 10, and 20 µg/mL) of sEVs on the proliferation of VEGF-stimulated HRECs. We found that sEVs (10 µg/mL) significantly inhibited the proliferation of HRECs in a concentration-dependent manner (Fig. 2B). We then compared the inhibitory capacity of the four groups, viz., PEDF, sEVs, a mixture of PEDF and sEVs without sonication, and PEDF-sEVs on the proliferation of ECs following VEGF stimulation. As expected, the strongest inhibition was observed in the PEDF-sEV group. In comparison, milder inhibition was observed in the PEDF, sEVs, and mixture groups (Fig. 2C).

Additionally, considering that angiogenesis requires the migration of ECs, we assessed whether PEDF-sEVs affected EC migration using Transwell and wound scratch assays. In the Transwell assay, PEDF, sEVs, and the mixture groups showed significant inhibitory effects on the migration of HRECs, whereas PEDF-sEVs showed a more marked inhibitory effect (Fig. 2D, E). Consistently, PEDF-sEVs exhibited the strongest inhibition of wound closure (Fig. 2F, G).

We further investigated the anti-angiogenic effects using a tube formation assay. VEGF stimulation significantly increased the number of nodes, master conjunctions, and the tube length. These effects were successfully reduced by PEDF-sEV treatment, and the difference was highly significant compared to the PEDF, sEVs, and mixture groups (Fig. 2H, I).

Moreover, we found that PEDF-sEVs had strong anti-angiogenic effects on HRECs, which was not only better than that of PEDF or sEVs alone, but also better than that of the mixture of PEDF and sEVs. These results revealed that PEDF-sEVs greatly enhanced the anti-angiogenic effects of PEDF and sEVs.

### PEDF-sEVs suppress VEGF-downstream signalling in HRECs

Existing studies have shown that PEDF suppresses AKT activation as well as ERK activation in response to VEGF stimulation [36], which strongly influences the proliferation and migration of ECs. Thus, we performed a western blot assay to test whether PEDF-sEVs suppress VEGF-downstream signalling in HRECs. As reported previously, our findings confirmed that VEGF induced the phosphorylation of AKT and ERK. Pre-treatment with PEDF and sEVs suppressed VEGF-induced AKT phosphorylation in HRECs, whereas PEDF-sEVs showed a stronger reduction (Fig. 2J, K). In addition, PEDF and sEVs alone reduced ERK1/2 phosphorylation upon VEGF stimulation, whereas PEDF-sEVs showed a more pronounced suppressive effect (Fig. 2J, K).

### PEDF-sEVs suppress inflammatory cytokine expression in HRECs

**Fig. 3:**
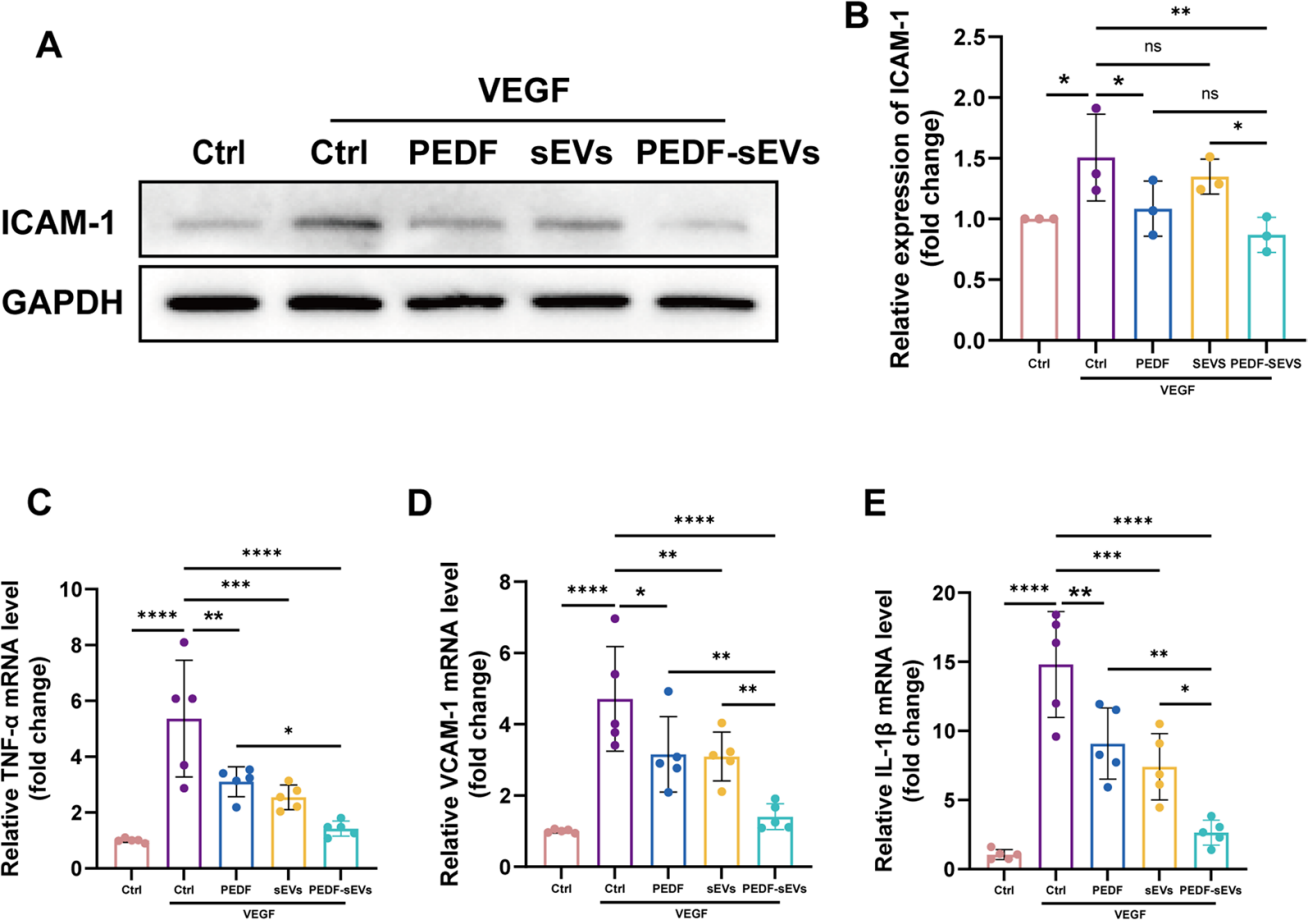
PEDF-sEVs suppress the expression of inflammatory cytokines in HRECs. **A** Starved HRECs were pre-treated with PEDF, sEVs, or PEDF-sEVs under stimulation with 10 ng/mL VEGF for 24 h. Representative western blot images showing the expression of ICAM-1 in HRECs. **B** Relative expression of ICAM-1 compared with that of GAPDH (*n* = 3). **C**, **D**,** E**. PCR analysis of the expression of TNF-α, VCAM-1, and IL-1β in HRECs (*n* = 5). The data are represented as mean ± SEM. *p < 0.05, **p < 0.01, ***p < 0.001, ****p < 0.0001

Previous studies have shown that PEDF suppresses the induction of intercellular cell adhesion molecule-1 (ICAM-1) expression by VEGF [37]. As ICAM-1 is an important inflammatory factor strongly associated with the adhesion and migration of HRECs, we performed a western blot assay to detect the expression of ICAM-1 to evaluate whether PEDF-sEVs could inhibit the expression of inflammatory cytokines in HRECs. Consistent with the findings in a previous study [37], PEDF decreased VEGF-induced up-regulation of ICAM-1 in HRECs. This effect was more pronounced in cells treated with PEDF-sEVs (Fig. 3A, B). We also used qPCR to determine the expression of other key molecules that drive inflammation, such as tumour necrosis factor-α (TNF-α), vascular cell adhesion molecule-1 (VCAM-1), and interleukin-1β (IL-1β). Upon exposure to VEGF, these molecules were highly expressed at the mRNA level, and their expression was reduced by PEDF and sEVs. A more pronounced effect was observed after treatment with PEDF-sEVs (Fig. 3C, D, E). These results demonstrate that PEDF-sEVs suppress the expression of inflammatory cytokines in HRECs more effectively than do PEDF or sEVs alone.

### PEDF-sEVs suppress neovascularisation and vaso-obliteration of the retina in OIR mice

**Fig. 4:**
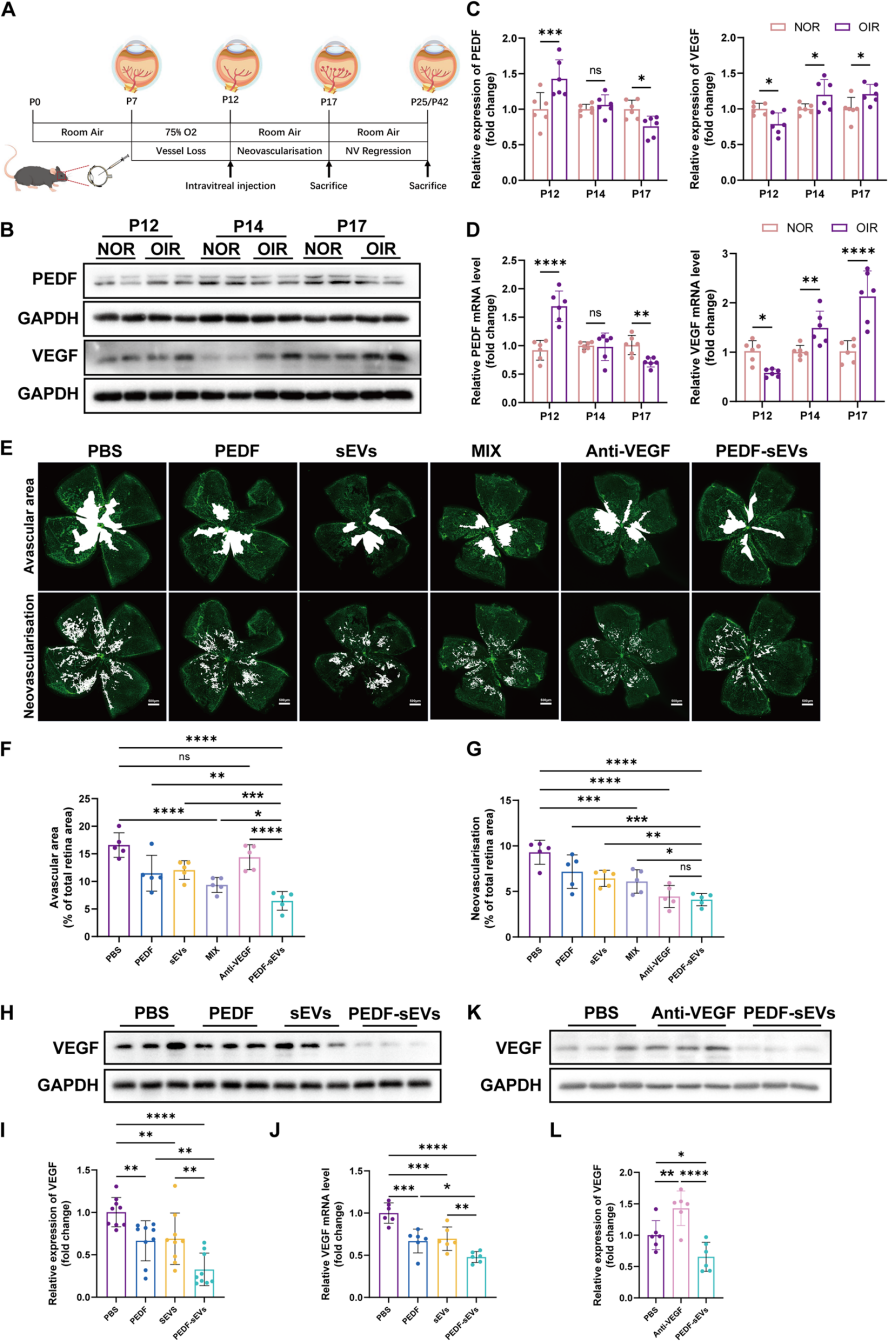
PEDF-sEVs suppress retinal neovascularisation and vaso-obliteration in OIR mice. **A** A schematic diagram showing the treatment of OIR mice with PBS, PEDF, sEVs, a mixture of PEDF and sEVs without sonication, anti-VEGF drugs, or PEDF-sEVs through intravitreal injection on P12. Pups were sacrificed and eyes were dissected on P17, P25, or P42. **B** The expression of PEDF and VEGF in the retinas of NOR and OIR mice on P12, P14, and P17. NOR refers to the normal C57BL/6J mice kept in room air that have not undergone hyperoxia. **C** Relative expression of PEDF and VEGF compared with that of GAPDH (*n* = 6 mice/group). **D** PCR analysis of PEDF and VEGF gene expression in the retinas of NOR and OIR mice on P12, P14, and P17 (*n* = 6 mice/group). **E** Representative confocal images of retinal vasculature stained with IsoB4 of OIR mice treated with PBS, PEDF, sEVs, a mixture, anti-VEGF drugs, or PEDF-sEVs on P17; scale bar = 500 μm. **F** Quantitative analysis of avascular area/total retinal area (*n* = 5 mice/group). **G** Quantitative analysis of neovascularisation/total retinal area (*n* = 5 mice/group). **H** VEGF expression in the retinas of OIR mice treated with PBS, PEDF, sEVs, and PEDF-sEVs on P17. **I** Relative expression of VEGF compared with that of GAPDH (*n* = 9 mice/group). **J** PCR analysis of VEGF gene expression in the retinas of OIR mice treated with PBS, PEDF, sEVs, and PEDF-sEVs on P17 (*n* = 6 mice/group). **K** VEGF expression in the retinas of OIR mice treated with PBS, anti-VEGF drugs, and PEDF-sEVs on P17. **L** Relative expression of VEGF compared with that of GAPDH (*n* = 6 mice/group). The data are represented as mean ± SEM. *p < 0.05, **p < 0.01, ***p < 0.001, ****p < 0.0001

The OIR mouse model is a widely used experimental disease model for ischaemic retinopathies and retinal neovascularisation that resembles ROP and certain aspects of proliferative diabetic retinopathy (PDR) [38]. Mouse pups were exposed to hyperoxic conditions (75% oxygen) from P7 to P12, as described previously [34] (Fig. 4A). As a result of hyperoxia, VEGF levels decreased and PEDF levels increased, leading to vaso-obliteration (VO) in the retina. When the pups were returned to ambient air, the relatively hypoxic environment caused the up-regulation of VEGF and down-regulation of PEDF on P14 and P17, resulting in pathological neovascularisation (Fig. 4B, C, D).

To test whether PEDF-sEVs inhibited angiogenesis in the OIR mouse model, pups were intravitreally treated with PEDF, sEVs, a mixture, anti-VEGF drugs, or PEDF-sEVs on P12. PBS was used as the control. Based on the analysis of the avascular areas of the retina, PEDF-sEVs had the most significant impact (66.96% ± 8.60% reduction) in restoring VO (Fig. 4E, F) among all groups. However, anti-VEGF treatment at a dose of 10 mg/mL, which is comparable to the human clinical dose, failed to rescue the non-perfusion area in the retina. According to the vessel tuft analysis, all treated groups exhibited a significant reduction in retinal neovascularisation compared with the control group. PEDF-sEVs demonstrated a stronger inhibitory effect on neovascularisation than did PEDF and sEVs in the OIR model, but showed a comparable inhibitory effect to anti-VEGF drugs (PEDF-sEVs 66.15% ± 8.52% reduction vs. Anti-VEGF drug 62.01% ± 10.92% reduction) (Fig. 4E, G).

Western blotting was used to assess the expression of VEGF in OIR retinas on P17. PEDF-sEVs significantly reduced the levels of VEGF compared with PEDF and sEVs alone (Fig. 4H, I, J). In addition, we observed that the levels of VEGF in retinas treated with anti-VEGF drugs was higher than that in the retinas of OIR mice (Fig. 4K, L). These results suggest that directly targeting VEGF results in a compensatory up-regulation of angiogenic cytokines, which has been previously observed in mouse and rat OIR models [13, 39].

In conclusion, we found no difference in the inhibition of neovascularisation between anti-VEGF drugs and PEDF-sEVs. Additionally, these data revealed that PEDF-sEVs significantly reduced retinal VO in OIR mice more effectively than did anti-VEGF drugs.

### PEDF-sEVs are superior to anti-VEGF drugs in suppressing retinal inflammation in OIR mice

**Fig. 5:**
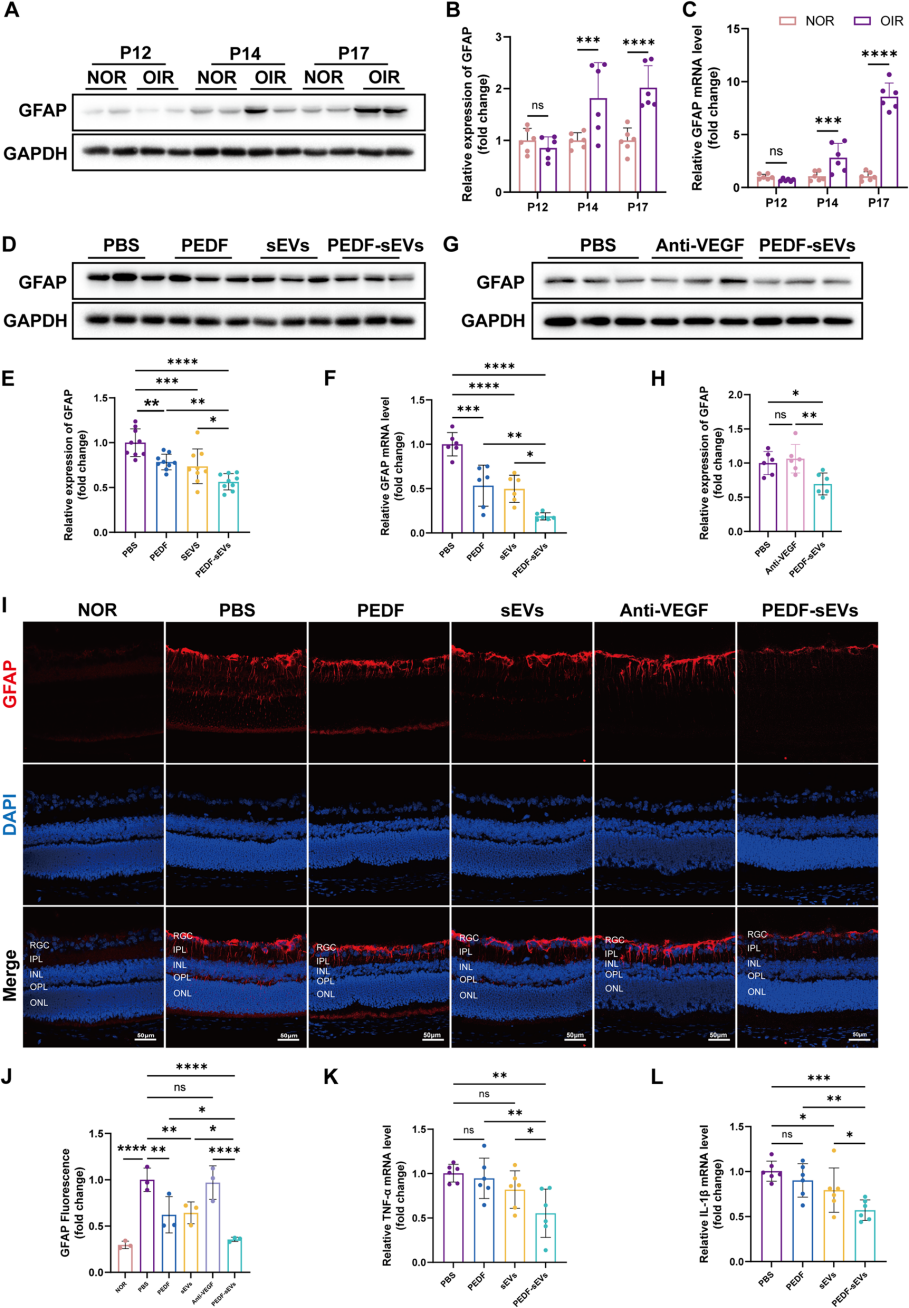
PEDF-sEVs suppress retinal inflammation in OIR mice. **A** GFAP expression in the retinas of NOR and OIR mice on P12, P14, and P17. **B** Relative expression of GFAP compared with that of GAPDH (*n* = 6 mice/group). **C** PCR analysis of GFAP gene expression in the retinas of NOR and OIR mice on P12, P14, and P17 (*n* = 6 mice/group). **D** GFAP expression in the retinas of OIR mice treated with PBS, PEDF, sEVs, and PEDF-sEVs treated on P17. **E** Relative expression of GFAP compared with that of GAPDH (*n* = 9 mice/group). **F** PCR analysis of GFAP gene expression in the retinas of OIR mice treated with PBS, PEDF, sEVs, and PEDF-sEVs on P17 (*n* = 6 mice/group). **G** GFAP expression in the retinas of OIR mice treated with PBS, anti-VEGF drugs, and PEDF-sEVs on P17. **H** Relative expression of GFAP compared with that of GAPDH (*n* = 6 mice/group). **I** Representative immunofluorescence staining images of retinas showing GFAP expression in NOR and OIR mice treated with PBS, PEDF, sEVs, anti-VEGF drugs, and PEDF-sEVs on P17; scale bar = 50 μm. **J** The fluorescence intensity was measured by integrated density using the ImageJ software (*n* = 3 mice/group, 3 sections per mouse, at least 3 images per section were analysed and the values were averaged). **K**,** L** PCR analysis of TNF-α and IL-1β gene expression in the retinas of OIR mice treated with PBS, PEDF, sEVs, and PEDF-sEVs on P17 (*n* = 6 mice/group). The data are represented as mean ± SEM. *p < 0.05, **p < 0.01, ***p < 0.001, ****p < 0.0001. RGC: Retinal ganglion cell; IPL: inner plexiform layer; INL: inner nuclear layer; OPL: outer plexiform layer; ONL: outer nuclear layer; RPE: retinal pigment epithelium

OIR mice exhibited retinal glial activation and elevated levels of inflammatory cytokines [40]. We found that the expression of GFAP in the retinas of OIR mice was up-regulated after the pups were returned to ambient air on P14 and P17 (Fig. 5A, B, C). Furthermore, we detected the expression of GFAP using western blotting to assess retinal inflammation. The data revealed that GFAP expression was inhibited by PEDF and sEVs, but this effect was more obvious with PEDF-sEV treatment (Fig. 5D, E, F). However, anti-VEGF drugs failed to reduce the expression of GFAP (Fig. 5G, H). Consistent with this, immunofluorescence staining for GFAP demonstrated that it was highly expressed in OIR mice but was significantly suppressed in the retinas of PEDF-sEV-treated mice. PEDF and sEVs exhibited weak inhibitory effects (Fig. 5I, J). Additionally, qPCR analysis showed a significant reduction in the expression of inflammation-related molecules, such as TNF-α and IL-1β, in response to PEDF-sEV treatment, whereas no clear effect was seen with PEDF and sEVs alone (Fig. 5K, L). These results indicate that PEDF-sEVs suppressed retinal inflammation more effectively than anti-VEGF drugs in OIR mice.

### **PEDF-sEVs are superior to anti-VEGF drugs in protecting retinal structure and in ameliorating retinal function in OIR mice**

**Fig. 6:**
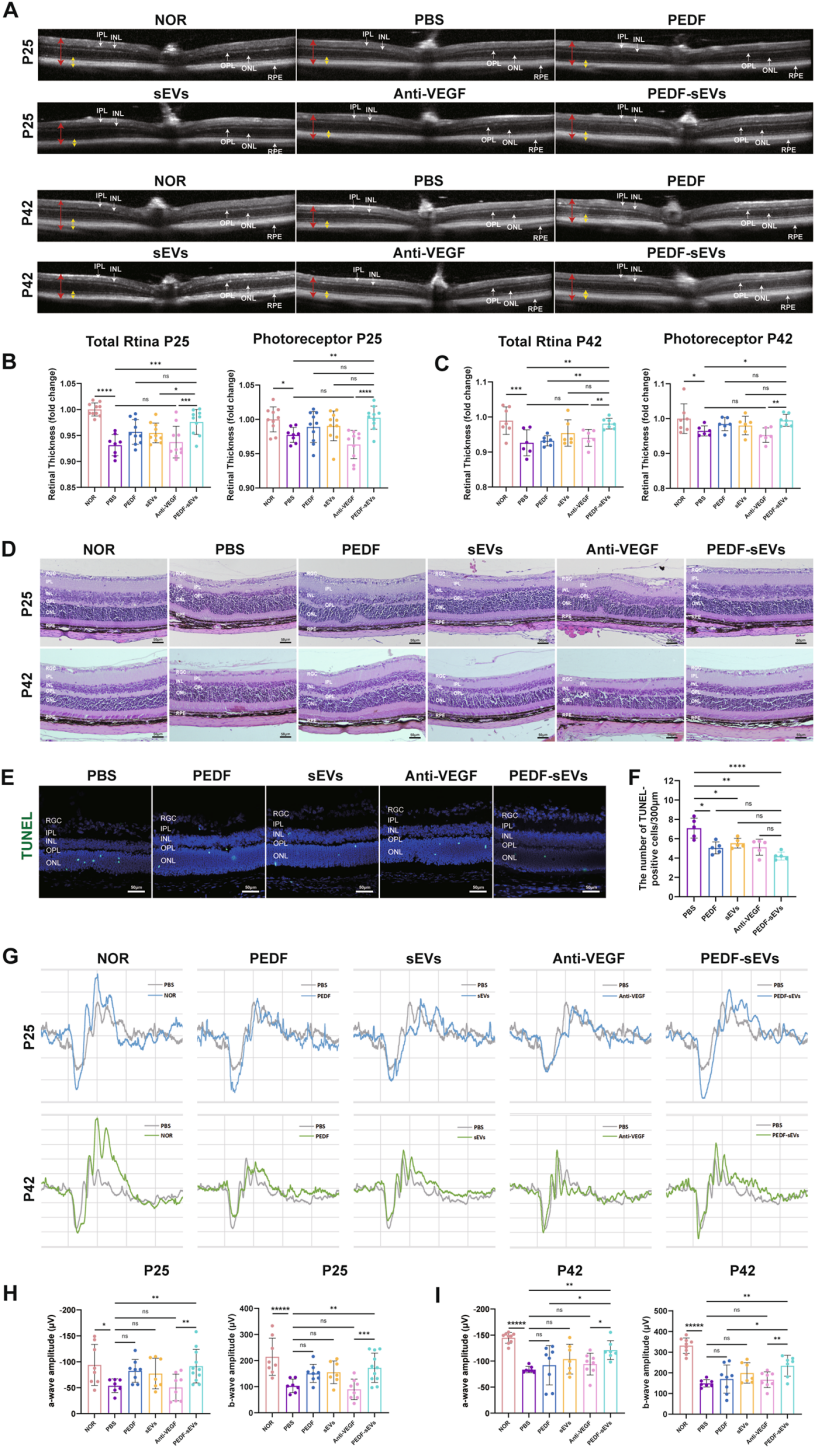
PEDF-sEVs are superior to anti-VEGF drugs in protecting retinal structure and in ameliorating retinal function in OIR mice. **A** Representative OCT images of NOR and OIR mice treated with PBS, PEDF, sEVs, anti-VEGF drugs, and PEDF-sEVs on P25 and P42. Red arrows indicate the total retina and green arrows indicate photoreceptor layer. **B** Quantitative analysis of total retina and photoreceptor layer thickness on P25 (*n* = 8–10 mice/group). **C** Quantitative analysis of total retina and photoreceptor layer thickness on P42 (*n* = 6–7 mice/group). **D** H&E staining of retinal tissues in NOR and OIR mice treated with PBS, PEDF, sEVs, anti-VEGF drugs, and PEDF-sEVs on P25 and P42 (*n* = 3 mice/group); scale bar = 50 μm. **E** Representative TUNEL assay images of eye sections of retinas from OIR mice treated with PBS, PEDF, sEVs, anti-VEGF drugs, and PEDF-sEVs on P17; scale bar = 50 μm. **F** Quantitative analysis of TUNEL-positive cells in the retinas (*n* = 5 mice/group, 5 sections per mouse, at least 5 images per section were analysed and the values were averaged). **G** Representative images of ERG response at a flash strength of 1.0 log (cd•s/ m^2^) in NOR and OIR mice treated with PBS, PEDF, sEVs, anti-VEGF drugs, and PEDF-sEVs on P25 and P42. **H** ERG analysis of a and b wave amplitudes on P25 (*n* = 7–11 mice/group). **I** ERG analysis of a and b wave amplitudes on P42 (*n* = 7–9 mice/group). The data are represented as mean ± SEM. *p < 0.05, **p < 0.01, ***p < 0.001, ****p < 0.0001. RGC: Retinal ganglion cell; IPL: inner plexiform layer; INL: inner nuclear layer; OPL: outer plexiform layer; ONL: outer nuclear layer; RPE: retinal pigment epithelium

Previous studies have shown that delayed retinal vascularisation may damage or alter the development of retinal structure and function [15]. OCT was performed on P25 and P42 to test the therapeutic effects of PEDF-sEVs on the disruption of the retinal structure in OIR mice. Compared with normal mice, the averaged OCT measurements in the PBS group showed a significant decrease for the thickness of total retina and photoreceptor layer on P25 (Fig. 6A, B). Significant improvement was observed in the PEDF-sEV-treated eyes, whereas no noticeable changes were observed in the eyes treated with anti-VEGF drugs. Consistent findings were noted on P42 (Fig. 6A, C).

The retinal structure was examined using H&E-stained sections. In contrast to normal mice, PBS-injected OIR eyes had a significant disruption of the outer plexiform layer (OPL) on P25 and P42 (Fig. 6D). Disruption of retinal morphology was significantly improved in eyes treated with PEDF-sEVs compared with those treated with PEDF, sEVs, or anti-VEGF drugs.

Likewise, TUNEL assays were used to test the toxicity of PEDF-sEVs to the retina. The results revealed that the number of TUNEL-positive cells in the retinas treated with PEDF-sEVs was lower than that in the retinas of OIR mice treated with PBS (Fig. 6E, F), indicating that the PEDF-sEV therapy was not toxic but exhibited neurotropic effects on the retinas.

To further evaluate the effect of delayed retinal vascularisation on retinal function, ERG was performed on P25 and P42. The amplitudes of the a- and b-waves in PBS-treated OIR mice were significantly decreased compared with those in normal mice on P25, with consistent performance observed on P42 (Fig. 6G, H, I). Analysis of the ERG response revealed that PEDF-sEVs had a significantly better therapeutic effect than either PEDF or sEVs on P25 and P42. In contrast, anti-VEGF drugs showed a trend towards deterioration.

In summary, eyes of PBS-treated OIR animals showed significant disruption of the retinal structure and function on P25 and P42. Compared with anti-VEGF drugs, PEDF-sEVs significantly improved the disruption in OIR mice, whereas an obvious disruption was observed in anti-VEGF drug-treated mice.

### **PEDF-sEVs effectively reduced the degradation of PEDF both in vitro and in vivo**

**Fig. 7:**
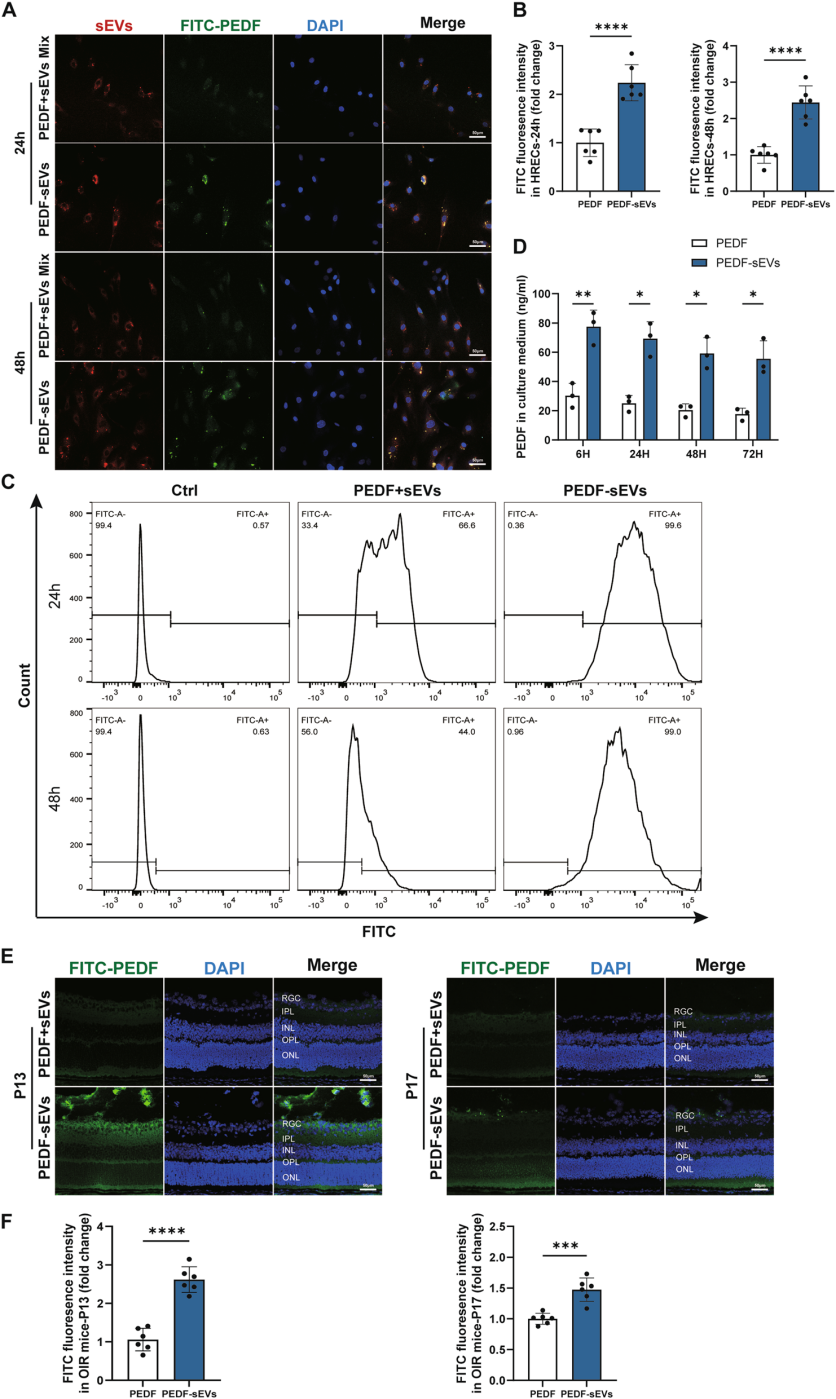
PEDF-sEVs effectively reduced PEDF degradation both in vitro and in vivo. **A** DiD-labelled sEVs and FITC-labelled PEDF were used to detect the distribution of PEDF and sEVs, respectively. PEDF-sEVs and a mixture of PEDF and sEVs without sonication were added to HRECs in culture. Representative confocal images show the cellular uptake of PEDF and sEVs in HRECs after 24 and 48 h; scale bar = 50 μm. **B** FITC fluorescence intensity in HRECs was measured by integrated density using the ImageJ software (*n* = 6/group). **C** PEDF-sEVs and a mixture were added to HRECs in culture. After 24 and 48 h, cells were collected for flow cytometry to analyse the cellular uptake efficiency of PEDF. **D** PEDF and PEDF-sEVs were added to HRECs in culture. After 6, 24, 48, and 72 h, the concentrations of PEDF in supernatants were measured using ELISA (*n* = 3/group). **E** Ocular distribution of PEDF and PEDF-sEVs in cryosections of OIR retinas on P13 and P17 after intravitreal injection on P12; scale bar = 50 μm. **F** FITC fluorescence intensity in cryosections of retinas was measured by integrated density using the ImageJ software (*n* = 6 mice/group; 3 sections per mouse, and at least 3 images per section were analysed and the values were averaged). The data are represented as mean ± SEM. *p < 0.05, **p < 0.01, ***p < 0.001, ****p < 0.0001. RGC: Retinal ganglion cell; IPL: inner plexiform layer; INL: inner nuclear layer; OPL: outer plexiform layer; ONL: outer nuclear layer; RPE: retinal pigment epithelium

According to our results, PEDF-sEVs were more effective than PEDF or sEVs alone in treating VEGF-induced ECs and OIR mouse model. However, there has been a lack of attention to the mechanisms by which sEVs enhance the treatment efficacy. Therefore, we used DiD-labelled sEVs and FITC-labelled PEDF to track sEVs and PEDF, respectively, and determine the distribution of PEDF-sEVs. HRECs were treated with PEDF-sEVs or a mixture of sEVs and PEDF without sonication for 24 or 48 h. Both DiD-labelled sEVs and FITC-labelled PEDF were taken up by HRECs. The mixture group displayed weak fluorescence signals, whereas the PEDF-sEV group showed strong FITC signals (Fig. 7A, B). Flow cytometry analysis also confirmed that the FITC-positive rate among HRECs in the PEDF-sEV group was 99%, whereas it was only 66% in the mixture group (Fig. 7C). In addition, the drug delivery system was stable for 48 h, with 99% FITC-positive HRECs in the PEDF-sEV group (Fig. 7C). The concentration of PEDF in the supernatants collected from the PEDF-sEV group was higher than that in the supernatants from the PEDF group at 6, 24, 48, and 72 h (Fig. 7D). These results indicate that PEDF-sEVs enhanced the therapeutic effect of PEDF by protecting it from degradation.

Similarly, following the intravitreal injection of PEDF or PEDF-sEVs, we dissected and sectioned the eyes 24 h or 5 d later (on P13 or P17) to assess the efficiency of PEDF-sEV delivery in the retinas of OIR mice. As evident from confocal images, the FITC signals were strongly enriched in the retinas injected with PEDF-sEVs, but were much weaker in the PEDF group (Fig. 7E, F). In addition, the FITC signals remained detectable and strong on P17, demonstrating high stability. In conclusion, PEDF-sEVs can be considered an effective drug delivery system because they significantly increase the stability and penetrability of PEDF.

## Discussion

In this study, we successfully obtained MSC-sEVs loaded with high concentrations of PEDF by sonication. We demonstrate that PEDF-sEVs have good stability and biosafety. PEDF-sEVs significantly inhibit pathological angiogenesis, inflammation, and neuronal degeneration. More importantly, PEDF-sEVs exerted better anti-inflammatory and neuroprotective effects than did anti-VEGF drugs in OIR mice. Our results strongly support the notion that PEDF-sEVs enhance the therapeutic effects by reducing the degradation and enhancing the penetrability of PEDF. Thus, PEDF-sEVs can provide an advantageous strategy for treating retinal pathological angiogenesis.

PEDF is a potent endogenous angiogenesis inhibitor. Homeostasis of PEDF and VEGF is critical for the development of retinal vasculature. Previous studies have found that PEDF levels decline in the vitreous and aqueous humour, and in the retina of patients with PDR [41–44]. Thus, restoring PEDF levels in the retina could be a potential strategy for the treatment of PDR. In a phase I clinical trial, PEDF showed therapeutic effects on wet AMD and appeared promising as an anti-angiogenic agent [45]. In addition, PEDF has neurotrophic, antioxidant, and anti-inflammatory properties in the retinas [19–21]. However, application of PEDF is limited because of its short half-life and low penetrability. To obtain a better and novel treatment, MSC-derived sEVs were used as carriers, and PEDF was successfully loaded into the sEVs.

Over the last few years, sEVs have received increased attention as drug carriers. Unlike other synthetic nanoparticles, the transmembrane and membrane-anchored proteins of sEVs can facilitate the transport of encapsulated contents and enable them to cross impermeable biological barriers, such as the blood–retinal barrier (BRB) and blood–brain barrier [46, 47]. Additionally, the intact bilipid membrane of sEVs protects the encapsulated PEDF protein from degradation, thus ensuring its long-term bioactivity [48, 49]. Considering that sEVs derived from MSCs have anti-inflammatory and neuroprotective effects, good biocompatibility, low immunogenicity, and a long half-life [46], we chose MSC-derived sEVs as carriers in this study. According to our results, MSC-sEVs alone had a positive anti-angiogenic, neurotrophic and anti-inflammatory effect. Using proteomic analysis, we previously found that these proteins contained in MSC-sEVs could be clustered into 43 biological processes including cell adhesion, immune response, cytoskeleton remodeling and development, and cell proliferation and differentiation, meanwhile, we found that there were 9 proteins that had anti-inflammatory, neuroprotective and anti-apoptotic effects [20].

In OIR mice, there are two types of retinal pathological blood vessels after the pups are returned to ambient air, namely VO and NV. Anti-VEGF drugs are widely known to inhibit retinal NV by blocking VEGF, but it has been observed that anti-VEGF treatments cannot diminish VO in OIR retinas [13, 14]. Consistent with previous studies, we found that anti-VEGF drugs failed to improve retinal non-perfusion areas in OIR mice, suggesting that targeting VEGF may block re-vascularisation of the ischaemic region. The dose of anti-VEGF treatment was comparable to the human clinical dose, which is 10 mg/mL. The volume of 1 µL was calculated based on the different size of human and mouse eyeballs. Notably, a significant reduction in retinal avascular areas was observed with the PEDF-sEV therapy, which is a potential advantage. VEGF also plays a critical role in neuroprotection and neurogenesis. VEGF simultaneously regulates neurogenesis and angiogenesis. Although the most obvious mechanism through which VEGF affects retinal neurogenesis is by increasing the blood flow to assist damaged tissues, it can also promote the survival of neuronal cells directly [50, 51]. Accordingly, VEGF is considered an important neuroprotectant and blocking it may directly affect neurodevelopment [15, 52]. We observed that the retinal neural function was diminished in OIR mice and there was no improvement with anti-VEGF therapy, which indicates that delayed retinal vascularisation caused by anti-VEGF drugs may lead to adverse effects on retinal function. Surprisingly, PEDF-sEVs showed marked effects on retinal structure and function in OIR mice, suggesting that PEDF-sEVs are effective nanotherapeutics for treating pathological angiogenesis in retinopathy.

Previous studies have shown that VEGF expression is closely associated with retinal inflammation. A recent study found that approximately 40% of patients with diabetic macular oedema (DME) failed to respond to anti-VEGF therapy because the up-regulation of inflammatory cytokines induced by VEGF could not be controlled merely by blocking VEGF [53]. We demonstrated that, unlike PEDF-sEVs, anti-VEGF drugs did not have any obvious anti-inflammatory effects in OIR mice. Despite anti-VEGF treatment having been used for over two decades, a more effective and safer therapy is needed considering its limitations.

PEDF-sEVs greatly enhanced the therapeutic anti-angiogenesis, anti-inflammation, and neuro-protective effect by enhancing the biological stability and penetrability of PEDF. In addition to sEVs protecting PEDF from degradation, we speculated that PEDF could also be secreted consistently into the extracellular space after PEDF-sEVs were endocytosed, thereby helping maintain PEDF at a high concentration through a stable sustained release. In addition, sEVs enhanced the biological penetration of PEDF, thereby maximising its effectiveness. Previous studies have shown that sEVs have anti-angiogenic, neurotrophic, and anti-inflammatory properties [20–22, 54]. Thus, a synergistic therapeutic effect was achieved when PEDF and sEVs were combined.

This study highlights the possible advantages of PEDF-sEVs over anti-VEGF drugs in the treatment of pathological neovascularisation. Although experimental results are promising, clinical applications are still far in the future. The limitation of this study is that, despite convincing results for significant protective effects of PEDF-sEVs in the OIR model, such a model cannot be used for long-term testing. Thus, it would be more judicious to conduct further research using animal models suitable for long-term treatment. In addition, the supply of MSC-sEVs remains extremely limited and far from meeting clinical needs. Further research is required to improve the production and purification of MSC-sEVs in order to promote the clinical application.

## Conclusions

In summary, our study demonstrated that PEDF-sEVs effectively enhanced the anti-angiogenic, anti-inflammatory, and neuroprotective effects of PEDF by increasing its stability and penetrability. Thus, PEDF-loaded MSC-sEVs could be a novel approach to treating retinal pathological neovascularisation.

### Supplementary Information

**Additional file 1: Table S1.** Primer sequences for qRT-PCR.

## Data Availability

All data generated or analysed during this study are included in this published article.

## Abbreviations

ROP: Retinopathy of prematurity
DR: Diabetic retinopathy
AMD: Age-related macular degeneration
RVO: Retinal vein obstruction
NV: Neovascularisation
PDR: Proliferative diabetic retinopathy
DME: Diabetic macular oedema
VEGF: Vascular endothelial growth factor
PEDF: Pigment epithelium-derived factor
sEV: Small extracellular vesicle
MSC: Mesenchymal stem cell
EC: Endothelial cell
HREC: Human retinal endothelial cell
ARVO: Association for research in vision and ophthalmology
RT: Room temperature
ERK: Extracellular signal-regulated kinase
GFAP: Glial fibrillary acidic protein
OCT: Optical coherence tomography
ERG: Electroretinogram
H&E: Haematoxylin and eosin
OIR: Oxygen-induced retinopathy
GAPDH: Glyceraldehyde-3-phosphate dehydrogenase
qPCR: Quantitative polymerase chain reaction
ICAM-1: Intercellular cell adhesion molecule-1
VCAM-1: Vascular cell adhesion molecule-1
TNF-α: Tumour necrosis factor-α
IL-1β: Interleukin-1β
BRB: Blood–retinal barrier
TUNEL: Terminal deoxynucleotidyl transferase-mediated dUTP nick end labelling
RGC: Retinal ganglion cell
IPL: Inner plexiform layer
INL: Inner nuclear layer
OPL: Outer plexiform layer
ONL: Outer nuclear layer
RPE: Retinal pigment epithelium
