# Supplementary material for: Enhanced therapeutic effect of PEDF-loaded mesenchymal stem cell-derived small extracellular vesicles against oxygen-induced retinopathy through increased stability and penetrability of PEDF
Source: J Nanobiotechnology. 2023 Sep 8;21:327. doi: 10.1186/s12951-023-02066-z (PMC10492320; doi:10.1186/s12951-023-02066-z)
Supplement: Supplementary file 1 — Additional file 1: Table S1. Primer sequences for qRT-PCR. [file 12951_2023_2066_MOESM1_ESM.docx]

**Table S1. Primer sequences for qRT-PCR**

| Gene | Sequence (5ʹ-3ʹ) |
| --- | --- |
| Mouse-GAPDH | F: CCTGTTGCTGTAGCCGTATTCA |
|  | R: CCAGGTTGTCTCCTGCGACTT |
| Mouse-TNF-α | F: CTCTTCTGTCTACTGAACTTCGG |
|  | R: AAGATGATCTGAGTGTGAGGGT |
| Mouse-IL-1β | F: AGTTGACGGACCCCAAAAG |
|  | R: AGCTGGATGCTCTCATCAGG |
| Mouse-PEDF | F: TCACCGGGCTCTCTACTACG |
|  | R: TGGTCCCATAGGACTTCTCCA |
| Mouse-VEGF | F: GCTACTGCCGTCCGATTGAG |
|  | R: CACTCCAGGGCTT CATCGTTA |
| Mouse-GFAP | F: ACCAGCTTACGGCCAACAG |
|  | R: CCAGCGATTCAACCTTTCTCT |
| Human-GAPDH | F: ATGGAAATCCCATCACCATCTT |
|  | R: CGCCCCACTTGATTTTGG |
| Human-TNF-α | F: CTGGGCAGGTCTACTTTGGG |
|  | R: CTGGAGGCCCCAGTTTGAAT |
| Human-VCAM-1 | F: AATGCCTGGGAAGATGGTCG |
|  | R: GATGTGGTCCCCTCATTCGT |
| Human-IL-1β | F: TTCGACACATGGGATAACGAGG |
|  | R: TTTTTGCTGTGAGTCCCGGAG |
